# A study of bovine mastitis, milking procedures and management practices on 25 Estonian dairy herds

**DOI:** 10.1186/1751-0147-48-22

**Published:** 2006-11-22

**Authors:** Laura Haltia, Tuula Honkanen-Buzalski, Irina Spiridonova, Arvi Olkonen, Vesa Myllys

**Affiliations:** 1 Department of Animal Diseases and Food Safety, Finnish Food Safety Authority Evira, Seinäjoki, Finland; 2 Faculty of Veterinary Medicine, University of Helsinki, Helsinki, Finland; 3 Department of Animal Diseases and Food Safety, Finnish Food Safety Authority Evira, Helsinki, Finland; 4 Estonian National Veterinary Laboratory, Tartu, Estonia; 5 Institute of Animal Husbandry of the Estonian Agricultural University, Tartu, Estonia

## Abstract

**Background:**

Mastitis prevalence, milking procedures and management practices were investigated in 25 big dairy herds supplying milk to an Estonian dairy company. The aim of the study was to provide information for the company to be used in their new udder health improvement program to be set up after the completion of this study.

**Methods:**

Quarter milk samples were collected from 3,166 cows for bacterial analysis and SCC (somatic cell counting). During the farm visit the veterinarian filled in a questionnaire about milking procedures and management practices with the help of farm managers. If the milk SCC of a cow or of a quarter exceeded 200,000/ml, the cow was defined as having mastitis.

**Results:**

The percentage of cows having inflammation in one or more quarters measured by SCC (200,000/ml) was 52.7%. *Corynebacterium bovis*, *Staphylococcus aureus *and coagulase negative staphylococci were the most common bacterial isolates. Women as farm owners, and participating in the milking, were associated with lower mastitis prevalence on the farm. Peat bedding was associated with higher mastitis prevalence.

**Conclusion:**

We demonstrated relatively high mastitis prevalence in this study. Contagious bacteria (eg. *S. aureus*, *C. bovis*, *S. agalactiae *and coagulase negative staphylococci) caused most of the infections. These infections are usually spread from cow to cow at milking if the milking hygiene is not good enough. The mastitis situation could be improved by improving milking procedures and hygiene.

## Background

Milk production is the most important branch of Estonian agriculture. Estonian milk industry faced major changes after the country gained independence in 1991 with the dissolution of the Soviet Union. Farming systems based on sovchoses and kolchoses have disappeared and they have been replaced by family holdings and big farms owned by companies. The abolition of sovchoses and kolchoses has also resulted in the bankruptcy of many dairy plants. The ability of small farms to invest in farm improvement is much more limited than that of bigger farms, resulting in differences between management practices, equipment and even in the feeding of cows.

After the political changes described above, few studies of mastitis have been carried out on Estonian dairy herds. *Tilga *and *Raid *examined 2,420 milk samples during the years 1988–1991, and found that 5% of Estonian cows had clinical mastitis and 15–30% subclinical mastitis [1]. Their criteria for a mastitic cow was ≥ 500,000 cells/ml in a milk sample. Staphylococci were the most prevalent pathogen (34%) in their study. *Klaassen et al*. studied somatic cell counts (SCC) of 9,220 cow milk samples of four different herds [2]. In their study, 7.8% of the cows had over one million cells/ml. *Aasmäe et al*. examined quarter milk samples having more than 400,000 cells/ml [3]. Udder pathogens were isolated from about one half of 157 samples from 30 farms. *Tilga and Raid *and *Klaassen et al*. did not specify sampling procedures. As the criteria for mastitis were different in the studies, the results are not comparable with each other. The sizes of the herds involved were not specified, nor were the criteria for the selection of individual animals.

The milk received by the dairy plant concerned in this study was classified in three quality categories in 1998: A/superior class with less than 415,000 cells/ml, B/first class with less than 521,000 cells/ml, and C/second class with less than 770,000 cells/ml. In 1998 the monthly average of superior-class milk delivered by the farms amounted to 96.3% with a variation range of 90.7%–100.0% [4]. The dairy plant started a project to improve udder health and farm hygiene in all herds supplying milk to the plant in 1998. The plant had no information on the udder health situation at the farms, and therefore a survey was considered necessary prior to the project. The dairy plant, processing 15 million litres of milk annually, is located in a typical Estonian farming area, about 25 km from Tartu. In addition to producing milk, the plant also produces yoghurt, cream, sour milk, "kefir" (sour milk) and "smetana" (thick sour cream). High-quality raw milk is essential to the production of these dairy products. The aim of the present study was to investigate mastitis prevalence, milking procedures and management practices in order to find out which measures should be taken into account in further udder health work.

## Materials and methods

### Study design

All farms (n = 25) producing milk for the dairy plant were selected for the study. The farms belonged to 12 owners. Between November 1998 and March 1999, a dairy advisor and a veterinarian collected quarter milk samples from all cows producing more than five litres of milk per day on one farm visit. Milk samples were collected from 3,166 cows. During the farm visit, the veterinarian filled in a questionnaire about milking procedures and management practices with the help of the farm managers. The questions concerned the breed and age of the herds, mean annual milk production, milking procedures, milking units/milker, installation year and maintenance of milking machines, measurement of vacuum, milking claw size, type of cowshed, bedding type and manure handling.

### Sampling

Sampling procedures and laboratory analyses were carried out as in a Finnish mastitis survey in 1995 [5,6]. Quarter milk samples were collected aseptically immediately before milking. The teat ends were cleaned with alcohol (70%) swabs and allowed to dry. The first few streams were discarded and the milk samples (about 5 ml) were collected in sterile 10 ml plastic tubes. Samples were immediately cooled and transported in cool bags to the Estonian National Veterinary Laboratories either in Tartu, Paide or Vöru. Another non-aseptic quarter milk sample of 40 ml was taken in a 40 ml plastic tube for SCC counts from each quarter. The samples were cooled and transported to the laboratory of the Institute of Animal Husbandry at the Estonian Agricultural University in Tartu.

### Analysis of milk samples

The SCC values of 12,328 quarter milk samples were measured by using the Fossomatic Milko Scan System 215 (Foss Electric, Hillerod, Denmark). A quarter was considered to have mastitis when the SCC was ≥ 200,000 [7,8]. Both subclinical and clinical cases are included in the results. To get a true average SCC of the farms, a value weighted by milk production was calculated from the analysed quarter milk samples by multiplying the average SCC of each cow by its milk production and then dividing the average results by the total milk production of each farm.

Microbiological analyses were carried out by streaking out 10 μl of milk with a sterile calibrated plastic loop on Trypticase Soy Agar plates (BBL, Cockeysville, MD, USA) containing 5% bovine blood. The plates were incubated at 37 ± 1°C for 48 hours. The plates were observed for bacterial growth after an incubation period of 24 and 48 hours. Bacterial species were identified according to *Honkanen-Buzalski *and *Seuna *[9].

### Data analyses

The association of milking procedures and management practices with mastitis prevalence and the prevalence of bacterial pathogens (listed in table 1) was analysed statistically at herd level. The prevalence of mastitis and the prevalence of each bacteria species in the herds were used as dependent variables and the data collected in the questionnaire were used as independent or categorical variables. Each variable was first analysed pairwise using t-test, the analysis of variance or correlation depending on the data structure. Their overall effect was further evaluated by analysing parameters using stepwise regression. Only the significant findings of regression analysis are reported. The statistical analysis was carried out with Statistix software (Analytical software, Tallahassee, USA).

**Table 1:** Bacterial findings during the survey.

Bacterial isolate	No of samples N = 11,640	% of all samples	% of isolates N = 3,581	SCC *10^3^/ml (SD)
*Streptococcus agalactiae*	110	0.9	3.1	3,263 (5,596)
*Streptococcus dysgalactiae*	68	0.6	1.9	2,576 (4,041)
*Streptococcus uberis*	86	0.7	2.4	2,431 (4,452)
Other streptococci, Enterococci	208	1.8	5.8	1,706 (3,100)
*Staphylococcus aureus*	752	6.5	21.0	1,026 (2,070)
Coagulase-negative staphylococci	568	4.9	15.8	508 (1,765)
*Corynebacterium bovis*	1,694	14.5	47.3	303 (956)
Coliforms	20	0.2	0.6	5,401 (5,423)
*Actinomyces *spp.	7	0.1	0.2	12,579 (9,262)
Other bacteria	68	0.6	1.9	565 (2,165)
No growth	8,059	69.2		221 (961)

The figures in the first two columns are the numbers and percentages of all quarter milk samples. The third column represents percentages of bacterial isolates. Mean SCC (SD) for each group are also presented.

## Results

### Somatic cell counts

**Figure 1:**
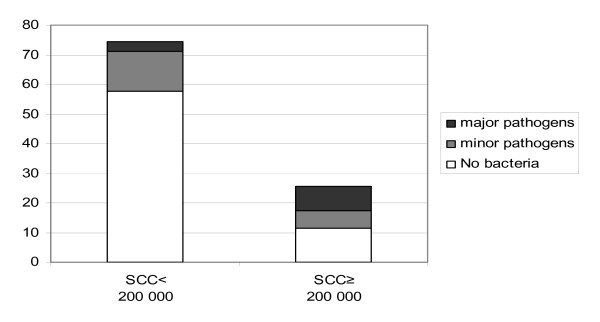
Distribution of healthy and mastitic udder quarters negative or positive for bacterial growth. Coagulase negative staphylococci *C. bovis *were considered as minor pathogens. Somatic cell counts (SCC) were measured by Fossomatic (n = 12,328).

The distribution of healthy and mastitic udder quarters with or without bacteria is shown in Figure 1. The proportion of quarters having SCC ≥200,000/ml was 25.3% (95% confidence interval 22.3 to 28.3) (Fig. 1). The percentage of cows having mastitis in one or more quarters, as measured by SCC, was 52.7% (95% confidence interval, 47.9 to 57.6). The average SCC calculated from quarter milk samples weighted by milk production was 370,120 cells/ml (95% confidence interval 313,970 to 426,270).

### Mastitis pathogens

The distribution of bacterial findings in 11,640 quarter milk samples, the proportions of bacterial isolates and the corresponding SCC are shown in Table 1. Samples with mixed cultures were excluded from these figures as these quarters cannot be categorised either as healthy or mastitic. *Corynebacterium bovis *was isolated in 14.6% of the samples and was the most common bacteria isolated (47.3% of isolates). *Staphylococcus aureus *was isolated from 6.5% of the samples and coagulase negative staphylococci from 4.9%. Streptococci were less frequent isolates.

### Herds, milk production and management procedures

The cow breeds in the investigated 25 herds were Estonian Red, and Estonian Holstein. Fourteen herds included both breeds, two herds contained only Estonian Red, and nine herds only Estonian Holstein. The average herd size was 164.4 cows, ranging from 15 to 463. The average age of the animals was 5.3 years. All herds are catalogued in the Estonian Animal Recording System. According to the data, the average production/cow was 5,520 kg/year. The latest bulk milk SCC value of the herds before the farm visit averaged 304,560 cells/ml (95% confidence interval 250,400 to 358,700), ranging from 69,000 to 730,000 cells/ml.

The animals were kept in tie-stall housing (88% of animals) at 22 farms, and only three herds had loose housing. Only two herds were not pasturing. The bedding material was sawdust in 32%, straw in 16%, cut straw in 4%, peat in 8% and a combination of the previously mentioned materials in 40% of the herds. All herds had solid manure removal. Peat bedding was associated with higher mastitis prevalence (p < 0.01).

The farms had seven different milking machine types. The age of the milking machines varied from one to 22 years. The milk claws were smaller than 150 ml in 16% of the milking machines. All milking machines had been serviced after 1996 and 80% of the milking machines had been vacuum tested during the previous year. The average number of milking units/milker was 3.6, with a minimum of two and maximum of 16. The average number of cows/milker was 64.2, with a minimum of 15 and maximum of 155. Only seven farms used teat-dipping. Women as farm owners, and participating in the milking, were associated with lower mastitis prevalence at the farms (p < 0.05). Other milking practices and some management procedures are described in Table 2.

**Table 2:** Milking procedures and management practices in the 25 dairy herds studied.

Herd variable	n	%
No of milking units/milker		
≤ 3	18	72
> 3	7	28
Gender of milker in farm		
Male only	1	4
Female only	21	84
both genders	3	12
Participation in the dairy education program 1998	20	80
Using teat dipping	7	28
Foremilk stripping	25	100
Using teat ointment	5	20
Teat cleaning with		
-water	14	56
-washing solution	7	28
-disinfectant solution	4	16

None of the variables were statistically associated with the prevalence of mastitis.

None of the above-mentioned properties or management procedures were statistically associated with the presence of certain bacteria.

## Discussion

The farms in this study did not represent the usual type of Estonian farms, and the results should be compared only with farming in larger herds. The mean annual milk production (5,701 kg) in the herds of this study was somewhat higher than the average production of cows in Estonia (4,766 kg). The mean age of the cows was seven months lower than the average age of Estonian cows. Herd sizes were bigger than the average in Estonia, where about 50% of the herds have less than 10 cows [10]. In this study, the mean size of herds was 164.4 cows and only three herds had less than 20 cows.

It is usually difficult to compare results of surveillance studies because of differences in the sample selection and the criteria of mastitis. This study was carried out in a similar fashion as mastitis surveys in Finland in which a threshold level of 300,000 cells/ml were used for mastitis as suggested by *Klastrup and Schmidt Madsen *[6,11,12]. New data indicate, however, that the SCC of a cow that is not infected with mastitis pathogens is usually < 200,000 cells/ml and therefore this threshold level was used in this study [7,8]. To compare these results with the Finnish studies, we calculated mastitis prevalence also with a threshold level of 300,000 cells/ml. Thus the percentage of cows having mastitis in one or more quarters was 43.5% (95% confidence interval, 39.3 to 47.3), which was slightly higher than in Finland where the corresponding figure was 38% (95% confidence interval 35.1 to 40.5) in 1995 and 31% (95% confidence interval 28.4 to 33.1) in 2001 [11].

The mean SCC value as calculated from individual quarter milk samples appeared higher than that measured from the bulk milk (p = 0.07). The reason for this may be the fact that milk from cows suffering from acute clinical mastitis may have been treated with antibiotics and the milk discarded. Therefore the SCC of tank milk can give too optimistic a figure of the herd mastitis situation if it is the only measurement criteria. In the study conducted by *Klaassen et al *study, only 7.8% exceeded one million cells/ml (cow sample), which could be explained by the fact that in their material, milk samples were only collected from milk supplied to dairy plants [2]. In this study, 20.7% of cows had more than one million cells/ml (the average of quarter milk samples).

*C. bovis *was the most common bacterial finding in the study, which is in line with the Finnish study by *Pitkälä et al*. [11]. *Honkanen-Buzalski et al*. showed that *C. bovis *infection levels can differ considerably between herds, and that *C. bovis *infections are most common in herds not using teat dipping [13]. In this study, only 7 out of 25 herds used teat dipping, which may explain the high prevalence of *C. Bovis*. However, statistically significant differences were not detected. In a study conducted by *Aasmäe et al*., *C. bovis *was only found in 0.6% of the samples, but milk samples having less than 300,000 cells/ml were not investigated [3]. As *C. bovis *infections are normally mild, they may remain undetected [14]. The mean SCC of quarters with *C. bovis *was 303,000/ml. However, only 27.4% of the quarters with *C. bovis *infection had SCC ≥200,000/ml. In quarters infected with major udder pathogens, the mean SCC was ≥ 1,000,000/ml. Staphylococcal infections, *S. aureus *and CNS, were as common in this study as in the study of *Aasmäe et al*. [3].

Contagious bacteria (eg. *S. aureus*, *C. bovis *and *S. agalactiae*) caused most of the infections in this study. These infections are usually spread from cow to cow at milking if the milking hygiene is not good enough. Therefore it is likely that the mastitis situation could be improved by improving milking procedures and hygiene. It is known that milking procedures and practices followed by milkers strongly influence the prevalence of mastitis in herds as well as the overall milk quality [8,15]. In some of the herds, a milker used more than three milking units simultaneously, which can further impair the milking routine. However, milking procedures such as teat cleaning, udder preparation before milking, and overmilking were not observed in this study.

The farms in this study had clearly defined, separate work tasks between personnel. Typically, it was only milkers who did the milking. Each milker had his/her own group of about 60–65 cows. The employees' job descriptions were of the rigid kind and did not contribute to the prevention of mastitis. In the few cases where women owners participated in the milking, the prevalence of mastitis was lowest. This finding could be in line with the finding of *Barnouin et al*., according to which a herdsman precise in his techniques was associated with the herd having low SCC [16]. Owners probably are more motivated to maintain precise milking techniques than hired personnel and therefore a clearer motivation-based approach for the responsibilities of employees and supervisors would improve the mastitis situation.

The finding that peat was associated with higher mastitis prevalence could be due to difficulties in keeping animals clean. This association has not been observed in some other studies. *Hovinen et. al*. observed that bedding material (peat, sawdust or straw) on the teat was cleaned almost completely in automatic milking herds, and *Peltola *found out that peat litter had no effect on the state of health of the animals or on the quality of the milk [17,18].

There were also other deficiences in housing conditions and milking machines, which should be corrected. In many herds, for instance, the stalls were too short for the cows of today. The milk claws were too small (less than 150 ml) in some herds [19]. Even though statistical analysis did not point out specific risk factors, such as milking claws being too small or the infrequent use of teat dipping, it can be assumed that by correcting these deficiencies, udder health and milk quality could also be improved.

## Conclusion

Relatively high mastitis prevalence was revealed in this study. *S. aureus*, *C. bovis*, *S. agalactiae *and coagulase negative staphylococci caused most of the infections. These bacteria are easily spread from cow to cow at milking. Interestingly, peat was found to associate with higher mastitis prevalence. These results suggest that the mastitis situation could be improved by improving milking procedures and hygiene. Furthermore, the farms had clearly defined, separate work tasks between personnel. Clearer motivation-based approach for the responsibilities of employees and supervisors would contribute to the prevention of mastitis.

## Competing interests

The author(s) declare that they have no competing interests.

## Authors' contributions

LH took part in all aspects of investigation including planning, microbiological laboratory work and drafting of the manuscript. She carried out the sampling. TH-B participated in planning the study and drafting the manuscript. IS participated in planning and performing microbiological laboratory work. AO coordinated somatic cell counting in laboratory. VM carried out the data analyses and participated in drafting the manuscript.

## References

[B1] Tilga V, Raid H (1994). Stafülokokilisest mastiidist veistel. Bovine staphylococcal mastitis. The Estonian veterinary review.

[B2] Klaassen M, Peterson K, Kihu J (1995). Udara tervisliku seisundi hindamine somaatiliste rakkude arvu (SRA) alusel 1 cm^3 ^piimas. Evaluation of the health status of the udder on basis of somatic cell count (SCC) in 1 cm^3 ^of milk. The Estonian veterinary review.

[B3] Aasmäe B, Kalmus P, Ööpik T, Klaassen E (2000). Mastiidipatogeenide tundlikkus antibiootikumide suhtes. Antimicrobial susceptibility of bovine mastitis pathogens. The Estonian veterinary review.

[B4] Mõlder M, Meijerei Laeva (1999). financial manager, personal communication. Tartu.

[B5] Honkanen-Buzalski, Sandholm M, Honkanen-Buzalski T, Kaartinen L, Pyörälä S (1995). Sampling technique, transportation and history. The bovine udder and mastitis.

[B6] Myllys V, Asplund K, Brofeldt E, Hirvelä-Koski V, Honkanen-Buzalski T, Junttila J, Kulkas L, Myllykangas O, Niskanen M, Saloniemi H, Sandholm M, Saranpää T (1998). Bovine mastitis in Finland in 1998 and 1995 – Changes in prevalence and antimicrobial resistance. Acta vet Scand.

[B7] Ruegg PL, Reinemann DJ (2002). Milk quality and mastitis tests. Bovine Pract.

[B8] Schukken YH, Wilson DJ, Welcome F, Garrison-Tikofsky L, Gonzales RN (2003). Monitoring udder health and milk quality using somatic cell counts. Vet Res.

[B9] Honkanen-Buzalski T, Seuna E, Sandholm M, Honkanen-Buzalski T, Kaartinen L, Pyörälä S (1995). Isolation and identification of pathogens from milk. The bovine udder and mastitis.

[B10] Agricultural register and information centre (1999). Results of animal recording in Estonia 1998. Estonia.

[B11] Pitkälä A, Haveri M, Pyörälä S, Myllys V, Honkanen-Buzalski T (2004). Bovine Mastitis in Finland 2001. J Dairy Sci.

[B12] Klastrup O, Schmidt Madsen P (1974). Nordiske rekommendationer vedrøende mastitundesøgelser af kiertelprøver. Nord Vet Med.

[B13] Honkanen-Buzalski T, Griffin T, Dodd F (1984). Observations on Corynebacterium bovis infection of the bovine mammary gland. I. Natural infection. J Dairy Res.

[B14] Honkanen-Buzalski T, Bramley AJ (1984). Observations on *Corynebacterium bovis *infection of the bovine mammary gland. II. Experimental infection. J Dairy Res.

[B15] Peeler EJ, Green MJ, Fitzpatrick JL, Morgan KL, Green LE (2000). Risk factors associated with clinical mastitis in low somatic cell count british dairy herds. J Dairy Sci.

[B16] Barnouin J, Chassagne M, Bazin S, Boichard D (2004). Management paractices from questionnaire surveys in herds with very low somatic cell score through a national mastitis program in France. J Dairy Sci.

[B17] Hovinen M, Aisla AM, Pyorala S (2005). Visual detection of technical success and effectiveness of teat cleaning in two automatic milking systems. J Dairy Sci.

[B18] Peltola I, Nielson VC, Voorburg JH, L'Hermite P (1986). Use of peat as litter for milking cows. proceedings of Odour prevention and control of organic sludge and livestock farming Seminar: 15–19 April 1985, Silsoe UK.

[B19] Manninen E (1995). Effect of milking and milking machine on udder health. the bovine udder and mastitis.

